# Supplementary material for: Anti-Helicobacter pylori Compounds of Sambucus williamsii Hance Branch
Source: Plants (Basel). 2025 Aug 17;14(16):2558. doi: 10.3390/plants14162558 (PMC12389104; doi:10.3390/plants14162558)
Supplement: Supplementary file 1 [file plants-14-02558-s001.zip › plants-3785736-supplementary.pdf]

Supplementary Information

Anti-*Helicobacter pylori* compounds of *Sambucus williamsii* branch

Woo-Jin Jeong [1,†], Dong-Min Kang [1,†], Atif Ali Khan Khalil [2,†], Bashu Dev Neupane [1], Seong-Joon Cho [1], Na-In Yang [3], Ki Hyun Kim [3,*], and Mi-Jeong Ahn [1,*]

[1] College of Pharmacy and Research Institute of Pharmaceutical Sciences, Gyeongsang National University, Jinju 52828, Republic of Korea; 8.27mm@gnu.ac.kr (W.-J.J.); kdm7105@gnu.ac.kr (D.-M.K.); neupanebashudev@gnu.ac.kr (B.D.N.); 2024210438@gnu.ac.kr (S.-J.C.)

[2] Department of Biotechnology, Yeungnam University, Gyeongsan 38541, Republic of Korea; atif.khalil7799@gmail.com (A.A.K.K.)

[3] School of Pharmacy, Sungkyunkwan University, Suwon 16419, Republic of Korea; amellia00@skku.edu (N.-I.Y.)

*Correspondence: khkim83@skku.edu (K.H.K.); amj5812@gnu.ac.kr (M.-J.A.); Tel.: +82-55-772-2425 (M.-J.A.)

†These authors contributed equally to this work.

Figure S1. Isolation of compounds **1**–**7** from *Sambucus williamsii* branch

Figure S2. The ESI-QTOF mass spectrum of compound **1**

Figure S3. The ^{1}H-NMR spectrum of compound **1** (CD_3OD, 400 MHz)

Figure S4. The ^{13}C-NMR spectrum of compound **1** (CD_3OD,100 MHz)

Figure S5. The ESI-QTOF mass spectrum of compound **2**

Figure S6. The ^{1}H-NMR spectrum of compound **2** (CD_3OD, 400 MHz)

Figure S7. The ^{13}C-NMR spectrum of compound **2** (CD_3OD, 100 MHz)

Figure S8. The ESI-QTOF mass spectrum of compound **3**

Figure S9. The ^{1}H-NMR spectrum of compound **3** (CD_3OD, 400 MHz)

Figure S10. The ^{13}C-NMR spectrum of compound **3** (CD_3OD, 100 MHz)

Figure S11. The ESI-QTOF mass spectrum of compound **4**

Figure S12. The ^{1}H-NMR spectrum of compound **4** (CD_3OD, 600 MHz)

Figure S13. The ^{13}C-NMR spectrum of compound **4** (CD_3OD, 150 MHz)

Figure S14. The ESI-QTOF mass spectrum of compound **5**

Figure S15. The ^{1}H-NMR spectrum of compound **5** (CD_3OD, 300 MHz)

Figure S16. The ^{13}C-NMR spectrum of compound **5** **(**CD_3OD, 100 MHz)

Figure S17. The ESI-QTOF mass spectrum of compound **6**

Figure S18. The ^{1}H-NMR spectrum of compound **6** (CD_3OD, 300 MHz)

Figure S19. The ^{13}C-NMR spectrum of compound **6** (CD_3OD, 100 MHz)

Figure S20. The ESI-QTOF mass spectrum of compound **7**

Figure S21. The ^{1}H-NMR spectrum of compound **7** (CD_3OD, 300 MHz)

Figure S22. The ^{13}C-NMR spectrum of compound **7** (CD_3OD, 100 MHz)

Figure S23. Preparative HPLC chromatograms for the isolation of compounds

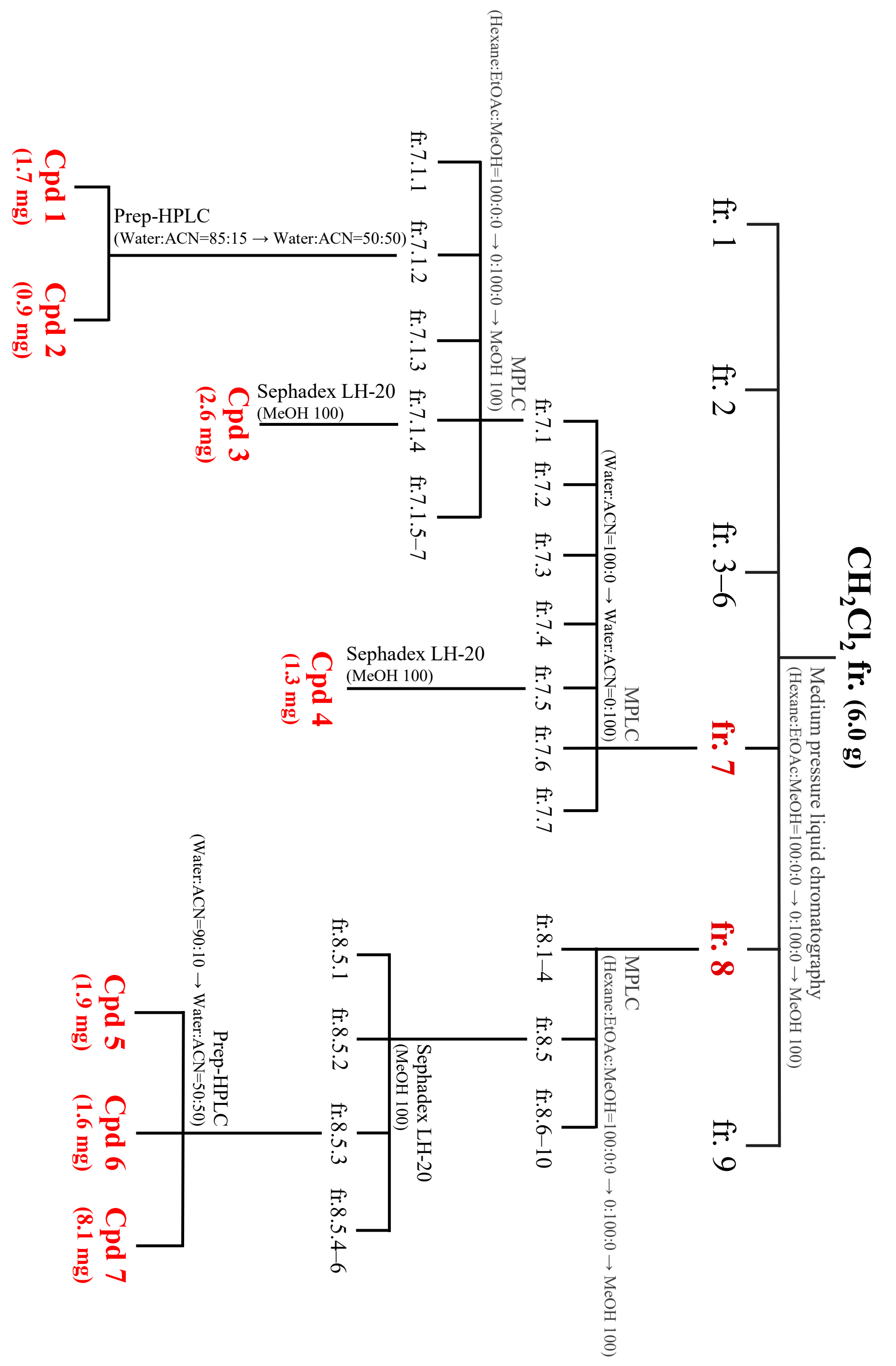

Figure S1. Isolation of compounds **1**–**7** from *Sambucus williamsii* branch

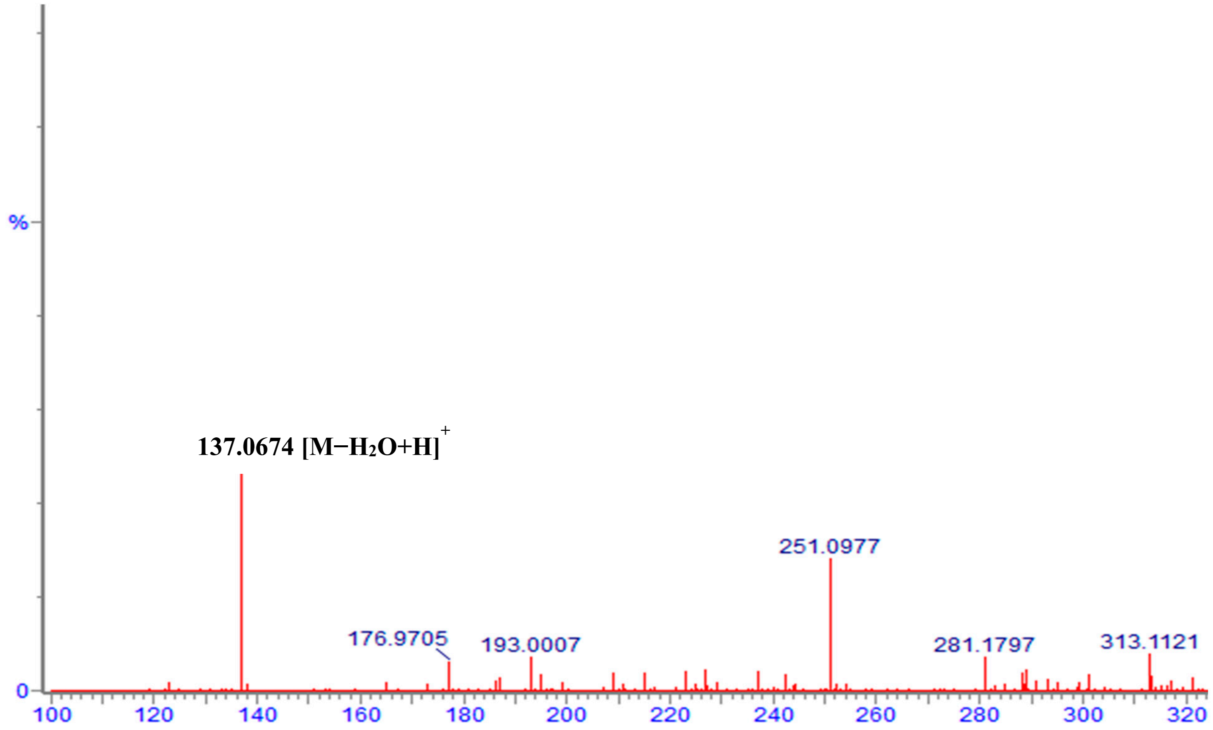

Figure S2. The ESI-QTOF mass spectrum of compound **1**

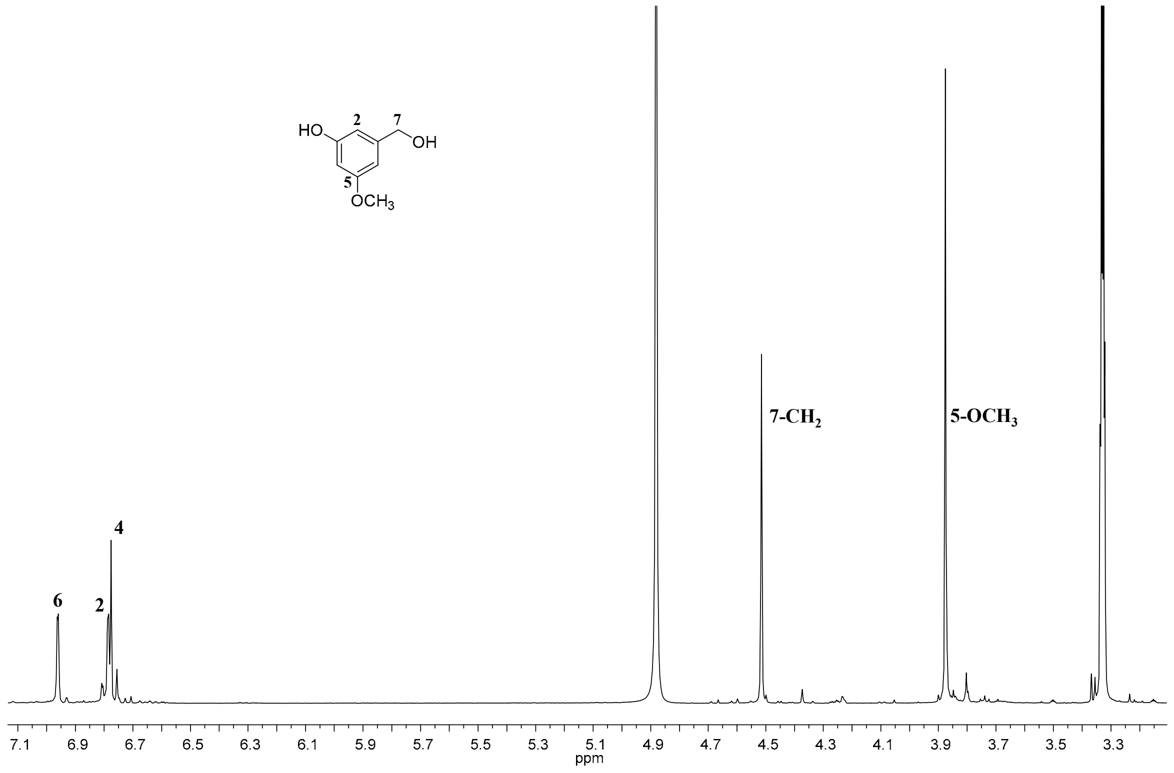

Figure S3. The ^{1}H-NMR spectrum of compound **1** (CD_3OD, 400 MHz)

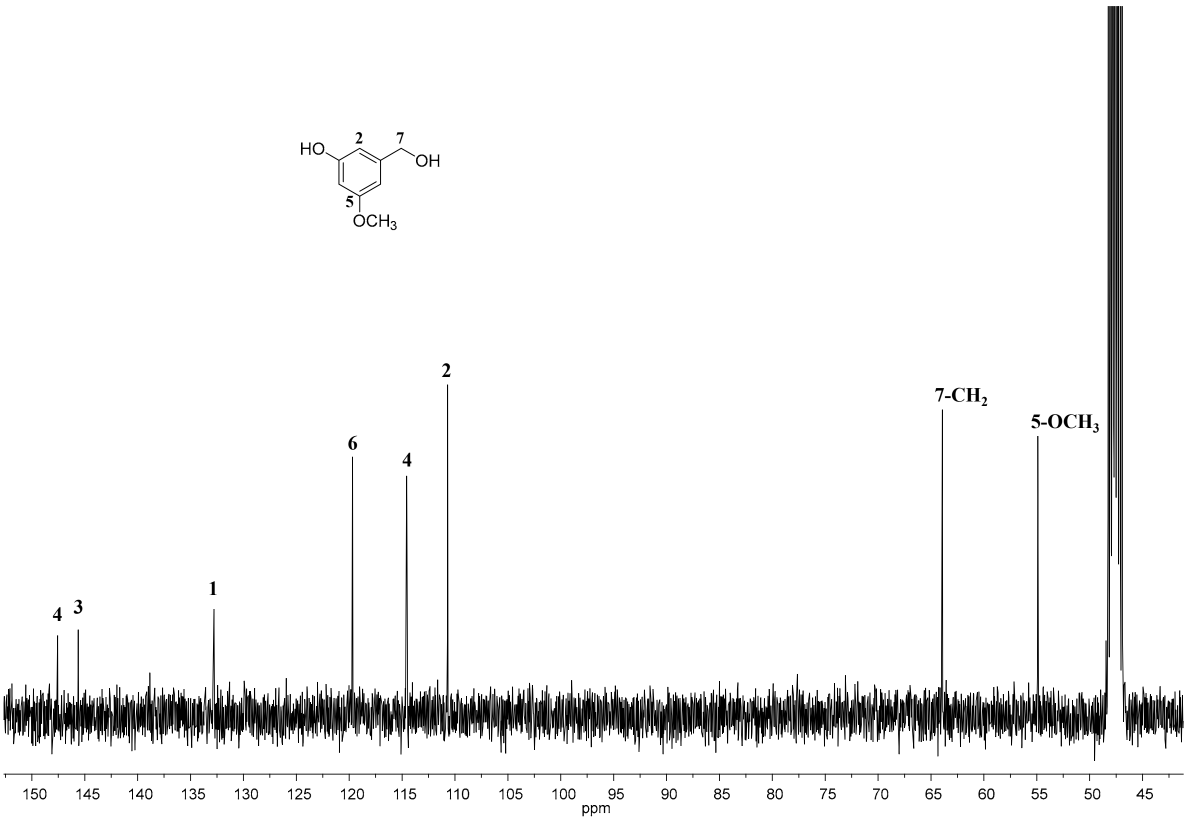

Figure S4. The ^{13}C-NMR spectrum of compound **1** (CD_3OD,100 MHz)

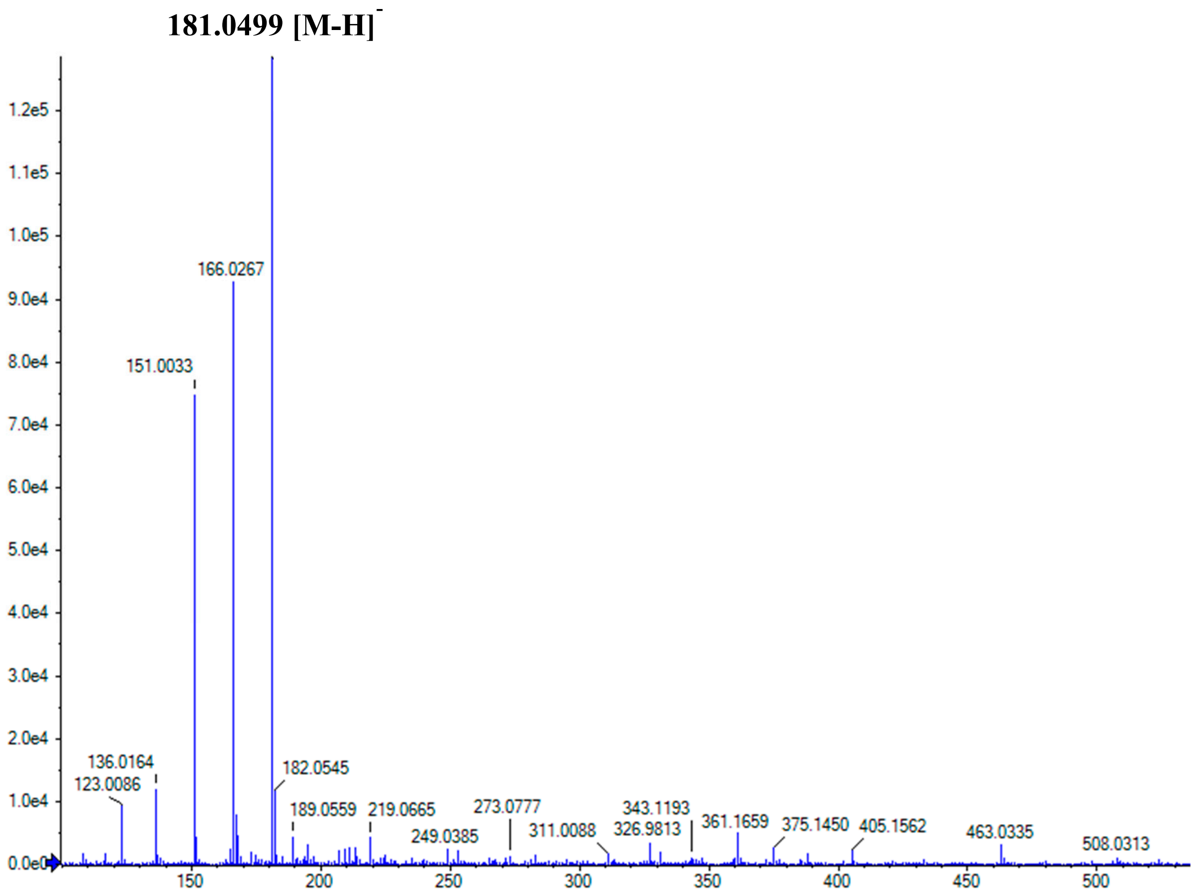

Figure S5. The ESI-QTOF mass spectrum of compound **2**

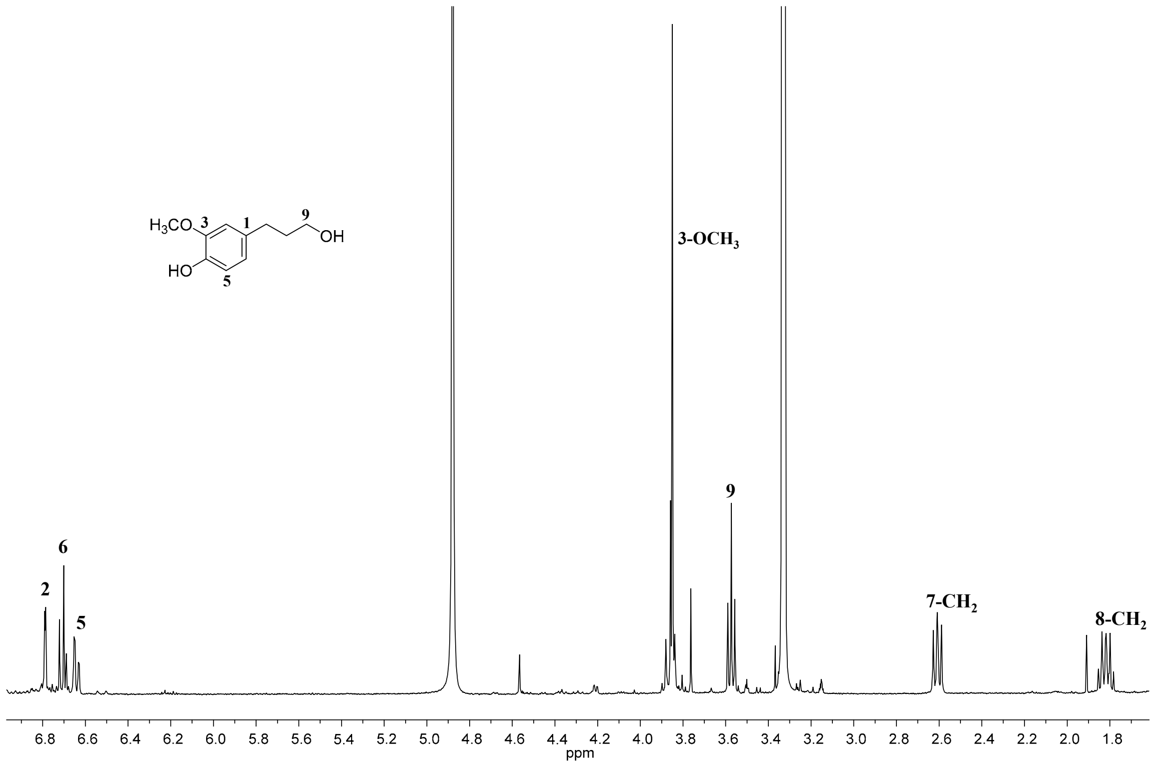

Figure S6. The ^{1}H-NMR spectrum of compound **2** (CD_3OD, 400 MHz)

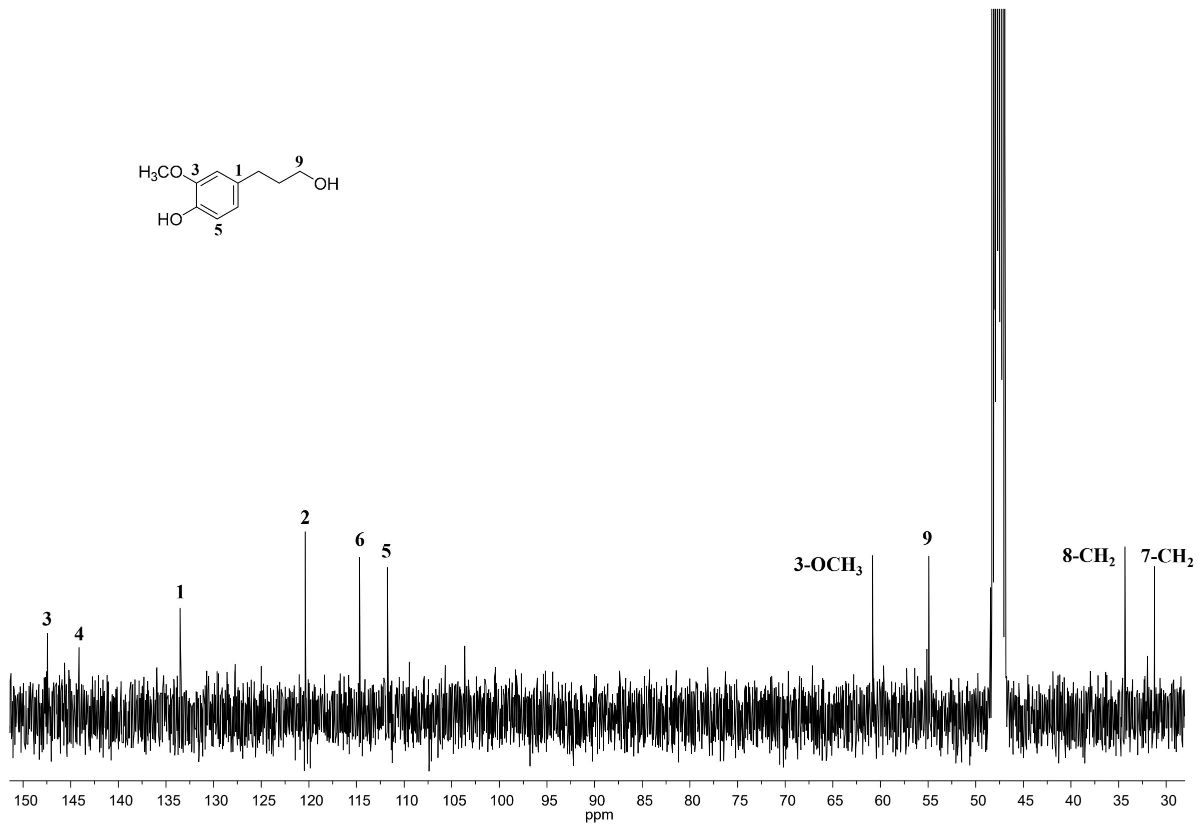

Figure S7. The ^{13}C-NMR spectrum of compound **2** (CD_3OD, 100 MHz)

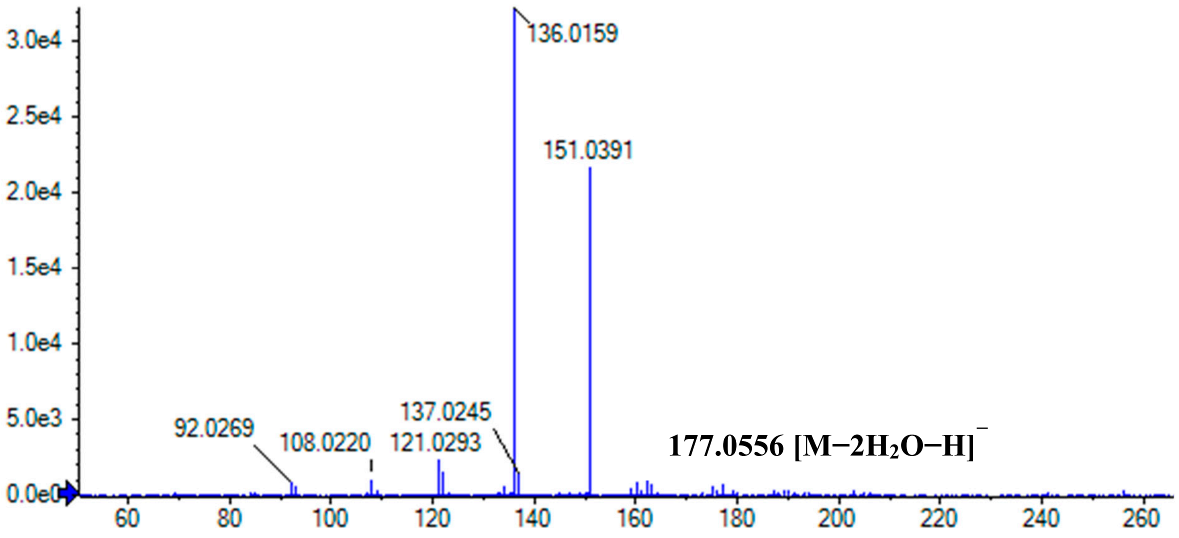

Figure S8. The ESI-QTOF mass spectrum of compound **3**

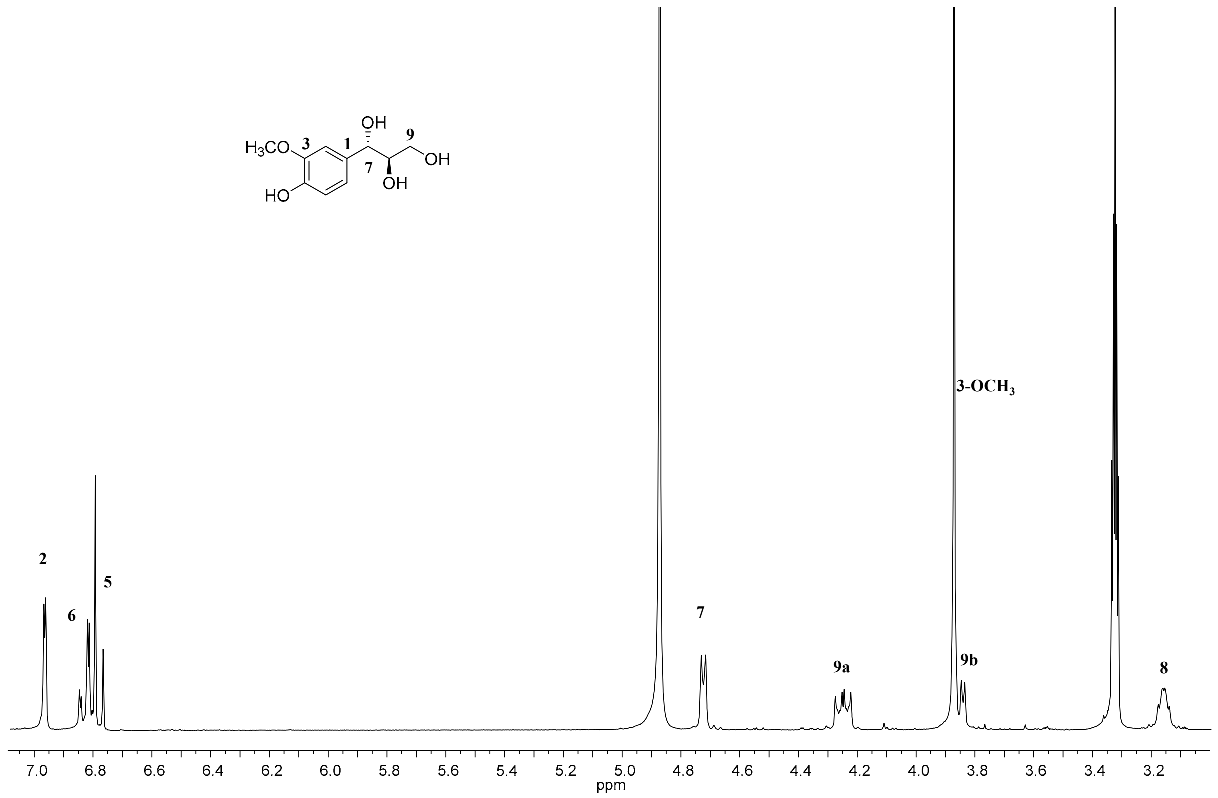

Figure S9. The ^{1}H-NMR spectrum of compound **3** (CD_3OD, 400 MHz)

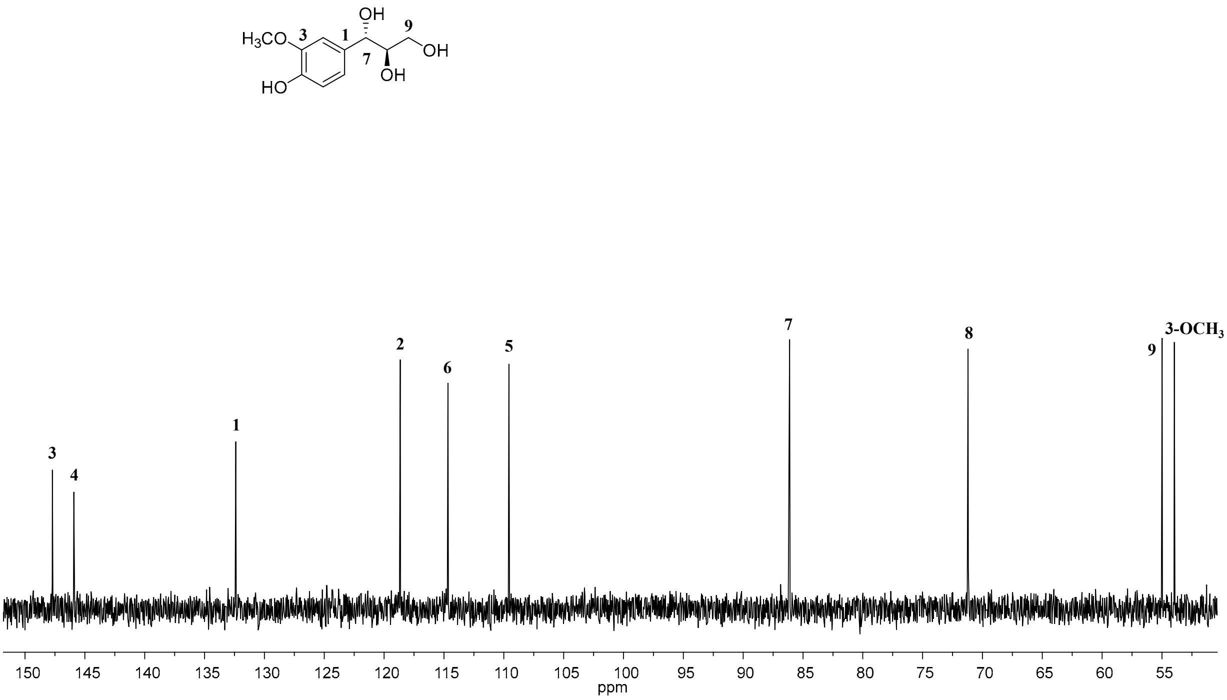

Figure S10. The ^{13}C-NMR spectrum of compound **3** (CD_3OD, 100 MHz)

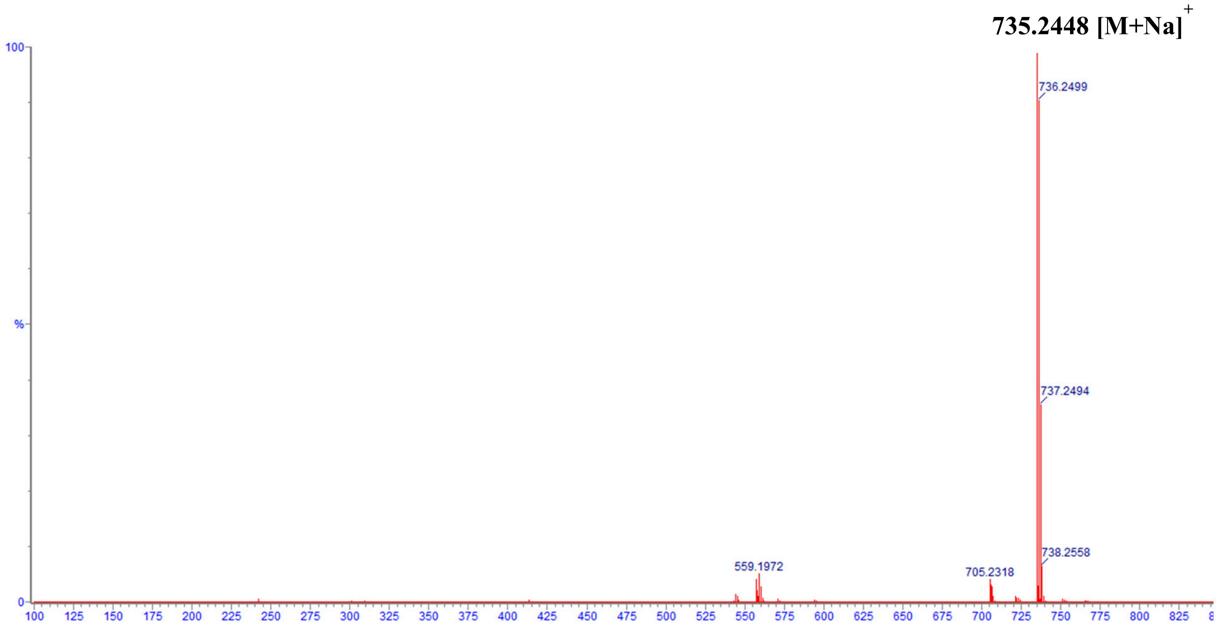

Figure S11. The ESI-QTOF mass spectrum of compound **4**

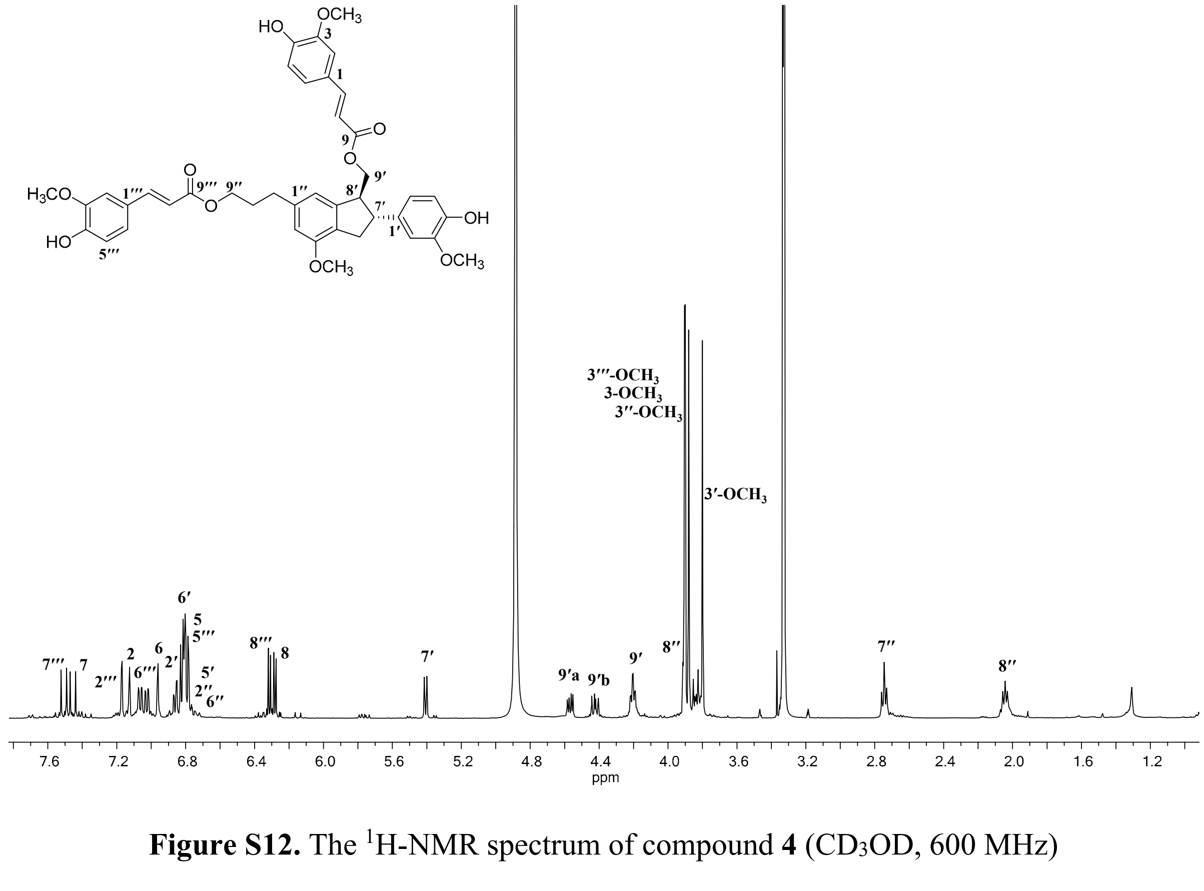

Figure S12. The ^{1}H-NMR spectrum of compound **4** (CD_3OD, 600 MHz)

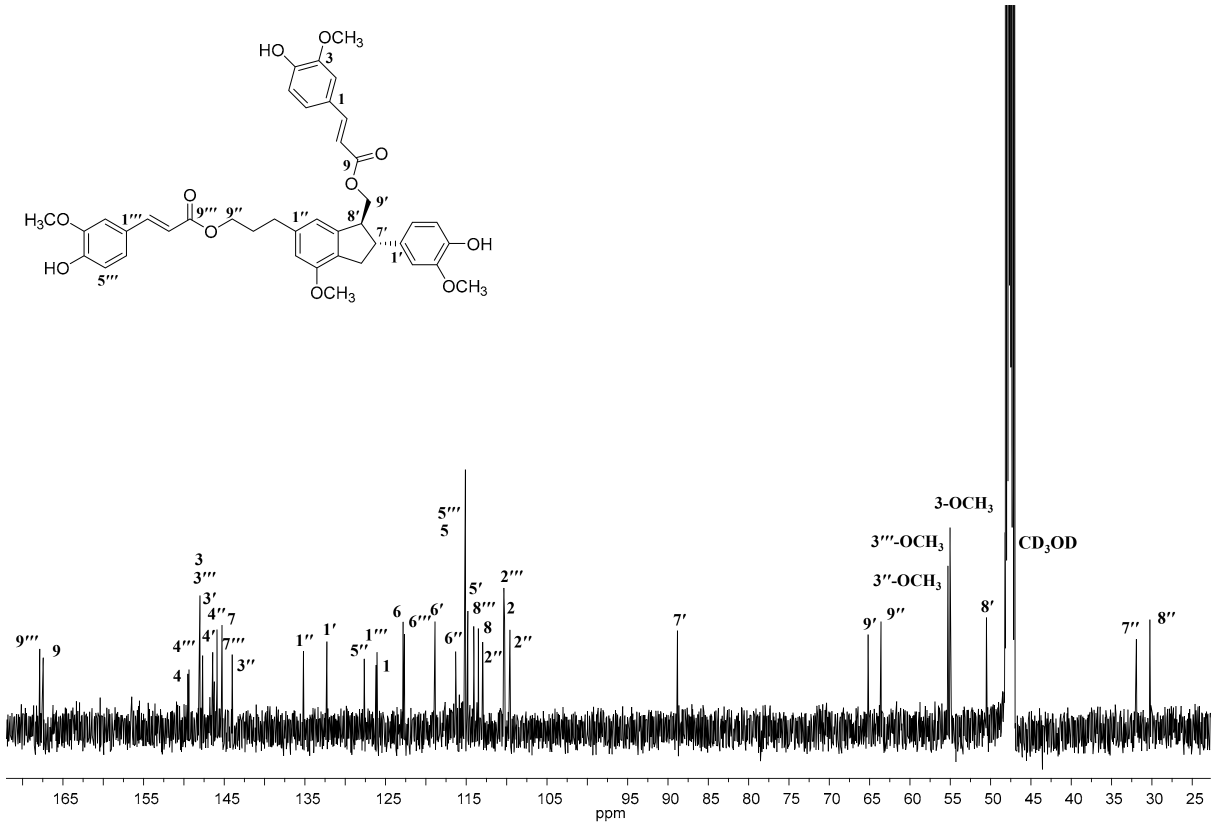

Figure S13. The ^{13}C-NMR spectrum of compound **4** (DMSO-*d*6, 125 MHz)

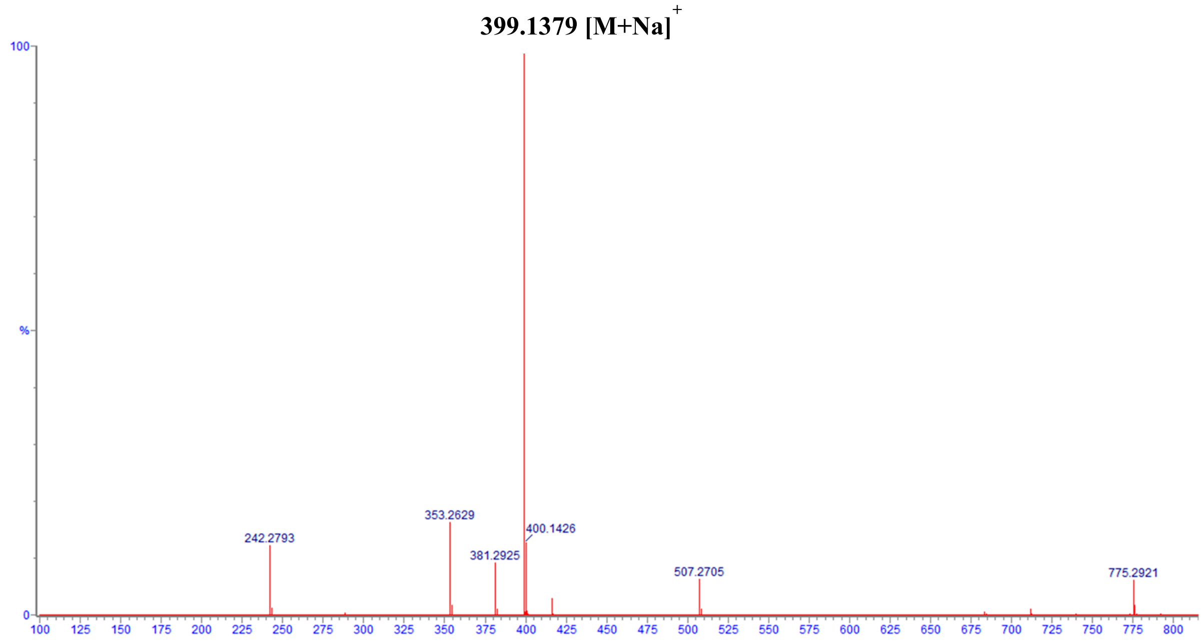

Figure S14. The ESI-QTOF mass spectrum of compound **5**

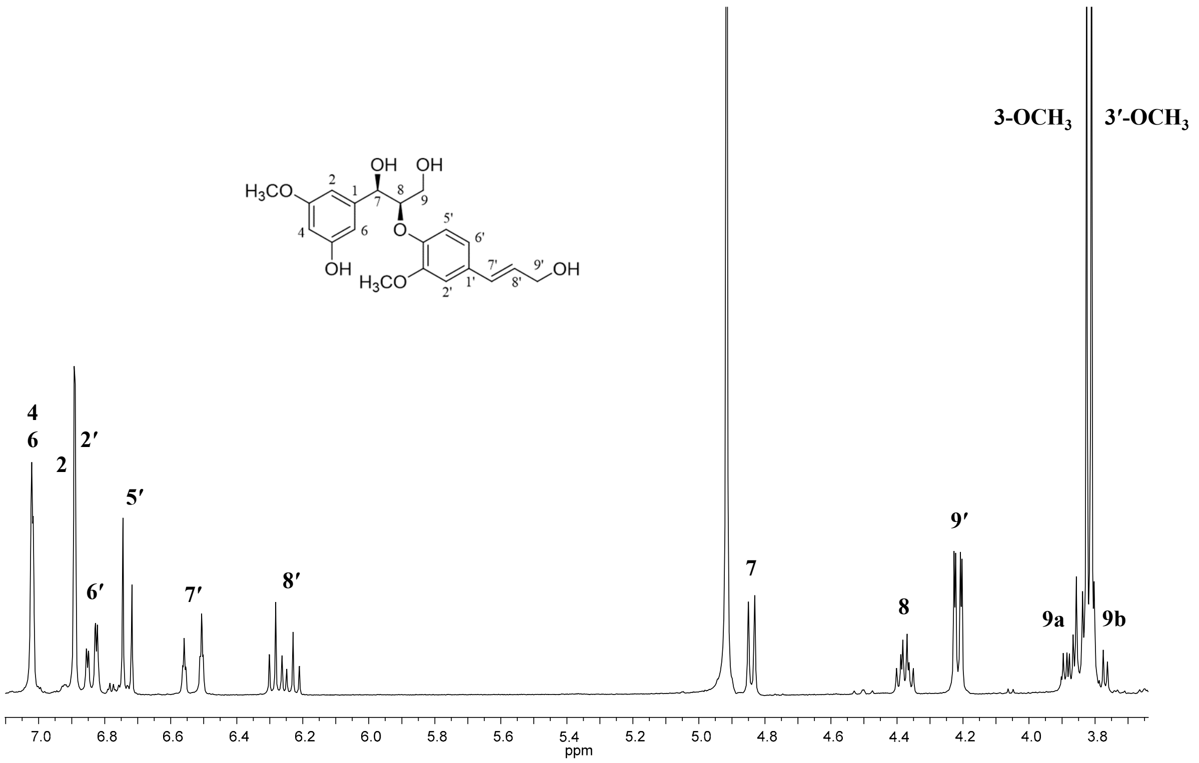

Figure S15. The ^{1}H-NMR spectrum of compound **5** (CD_3OD, 300 MHz)

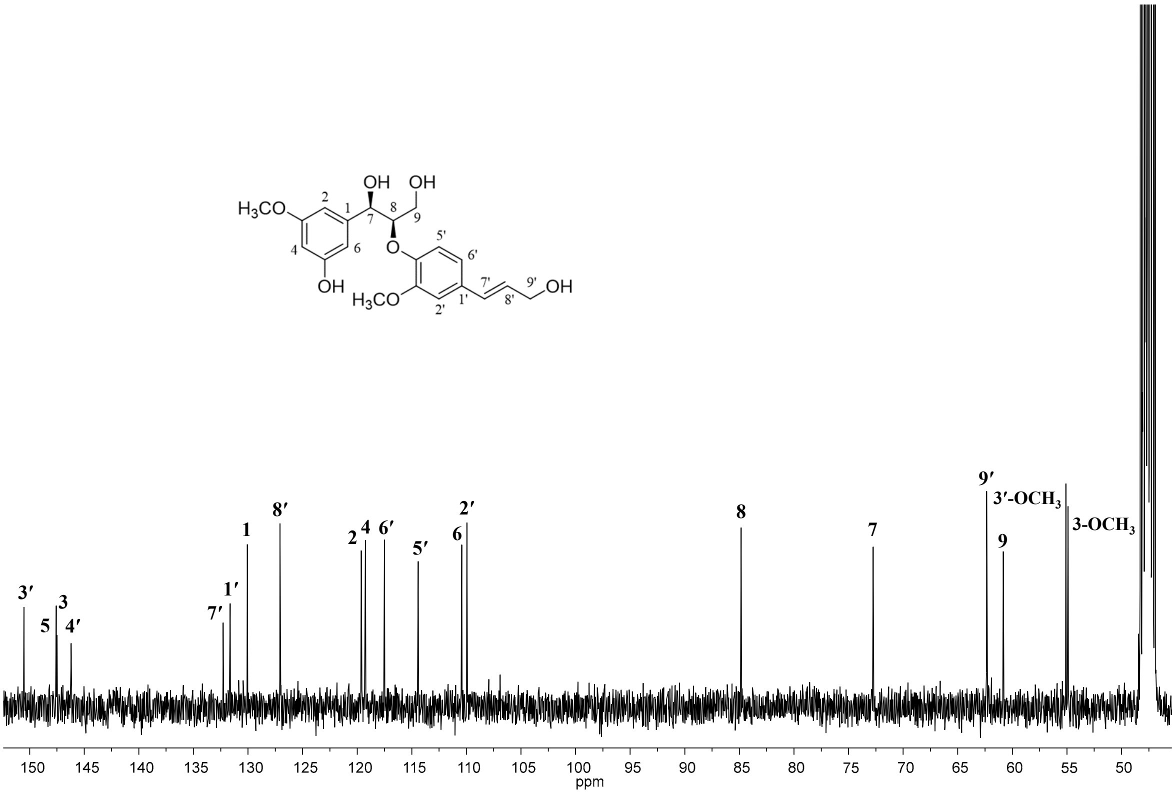

Figure S16. The ^{13}C-NMR spectrum of compound **5** (CD₃OD, 100 MHz)

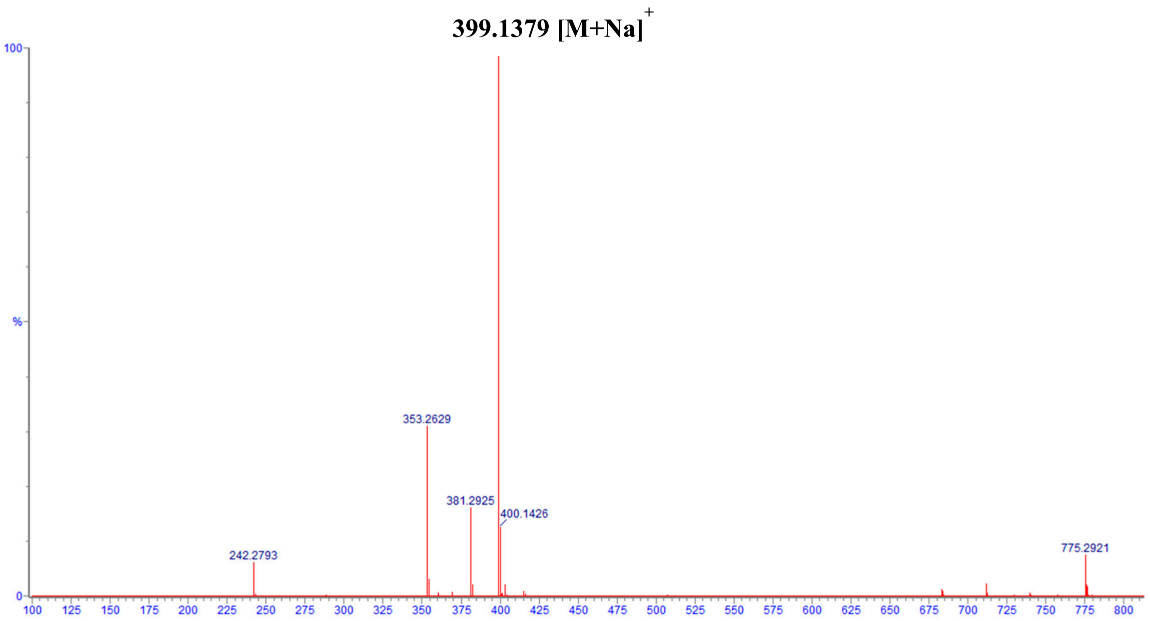

Figure S17. The ESI-QTOF mass spectrum of compound **6**

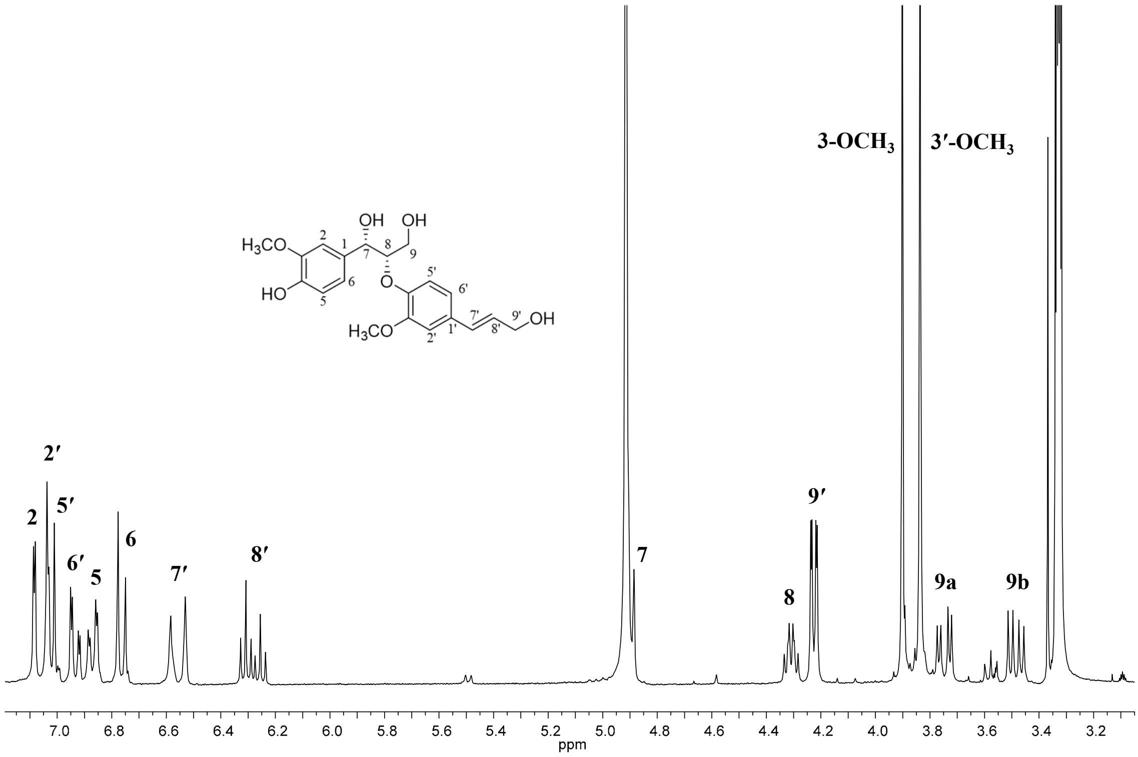

Figure S18. The ^{1}H-NMR spectrum of compound **6** (CD_3OD, 300 MHz)

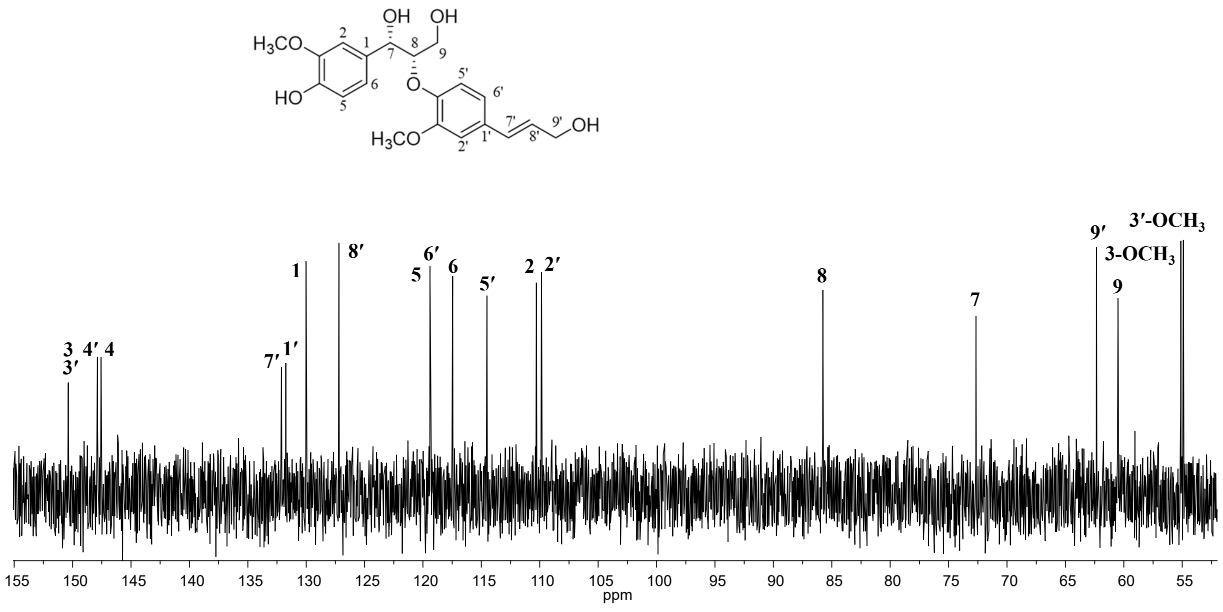

Figure S19. The ^{13}C-NMR spectrum of compound **6** (CD3OD, 100 MHz)

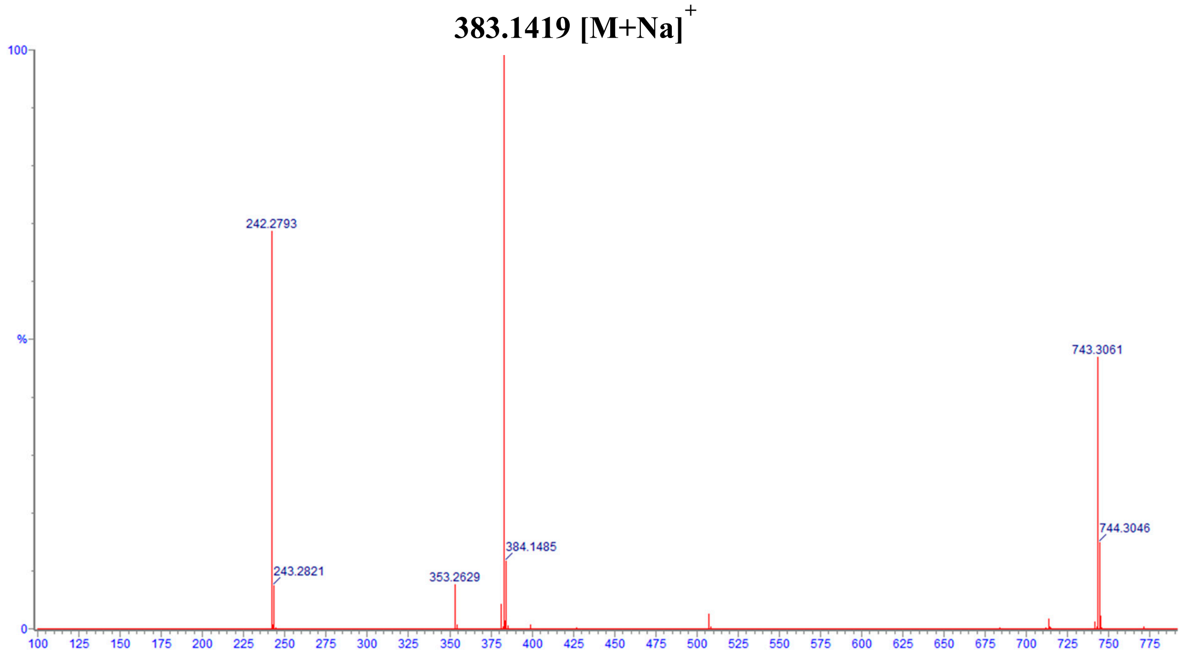

Figure S20. The ESI-QTOF mass spectrum of compound **7**

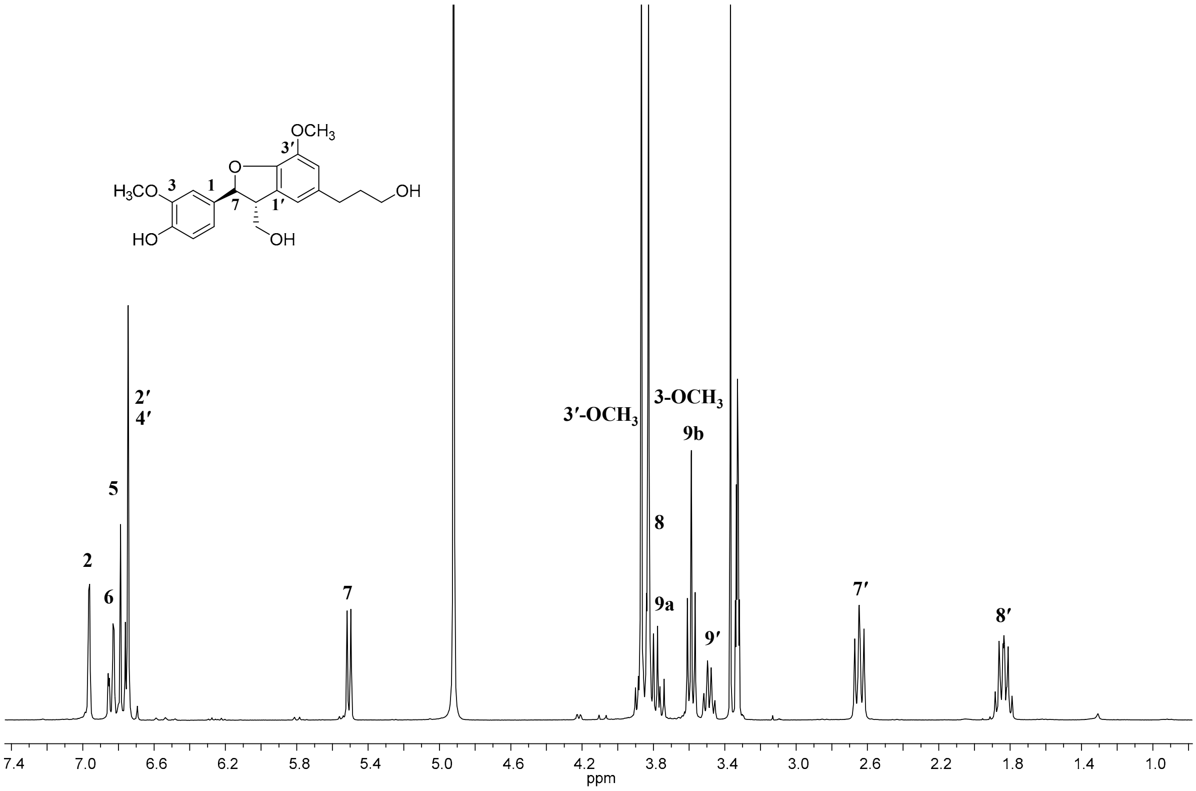

Figure S21. The ^{1}H-NMR spectrum of compound **7** (CD_3OD, 300 MHz)

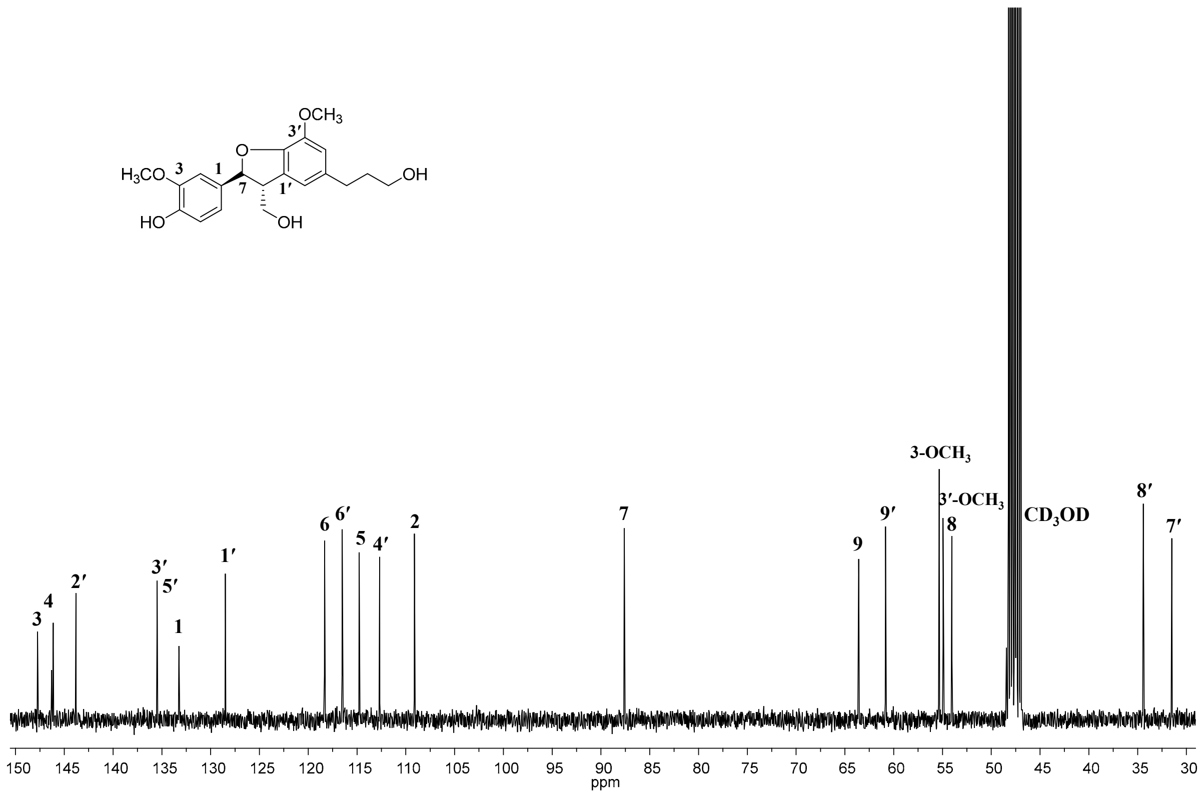

Figure S22. The ^{13}C-NMR spectrum of compound **7** (CD_3OD, 100 MHz)

Preparative HPLC conditions / 254 nm

- Column : Gemini C18 4.6 × 250 mm, 5 μm
- Wavelength : 254nm
- Column temp. : 30℃
- Flow late : 1 ml/min
- Solvent condition : (A) Water, (B) ACN

Times table		
min	A(%)	B(%)
0	85	15
5	85	15
40	50	50
50	0	95
Post time : 5 min		

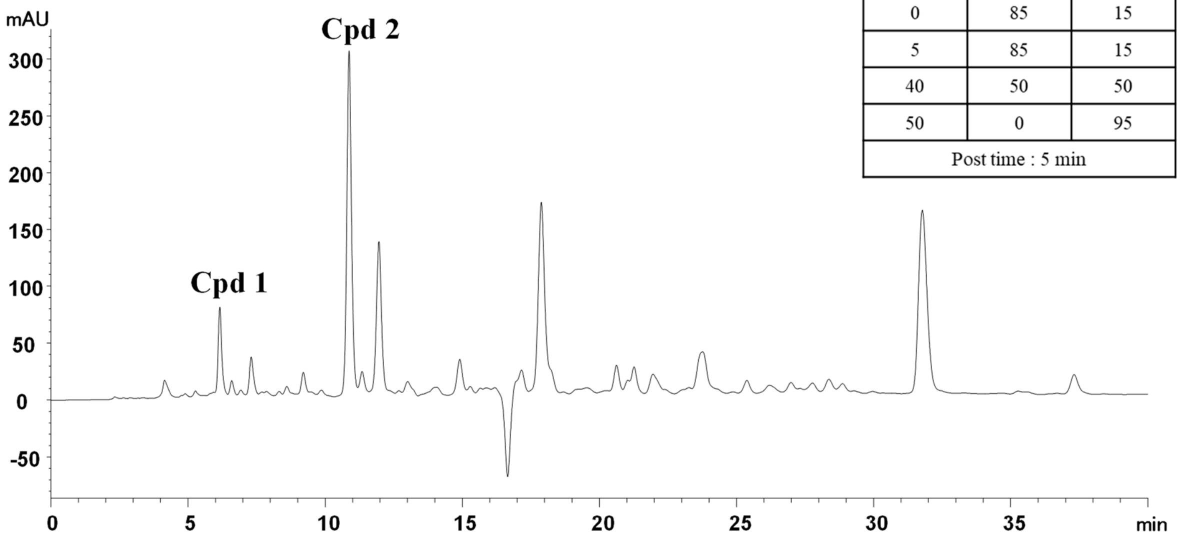

Times table		
min	A(%)	B(%)
0	90	10
5	90	10
40	80	20
80	50	50
90	5	95
Post time : 5 min		

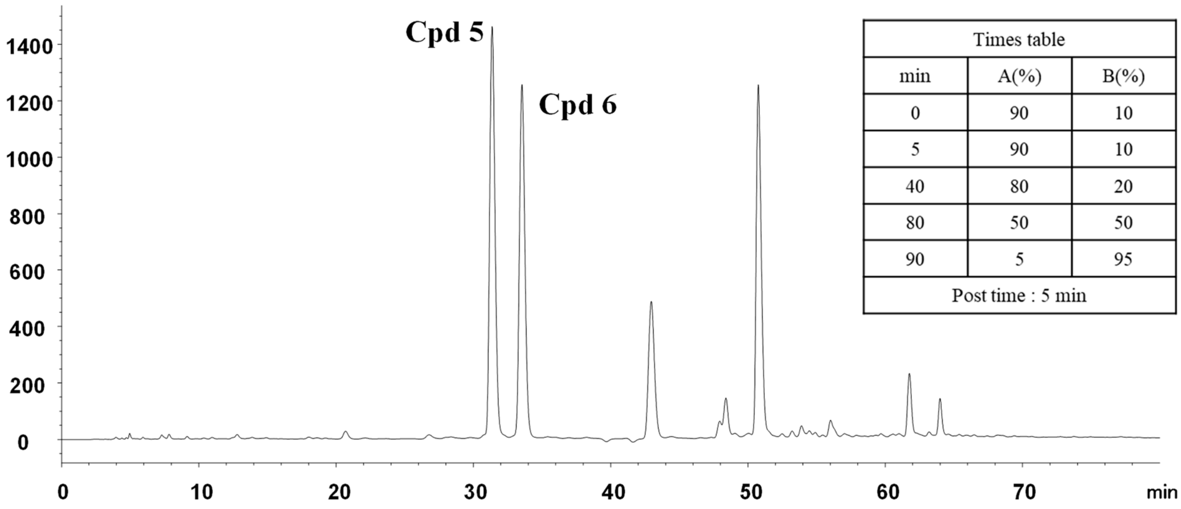

Figure S23. Preparative HPLC chromatograms for the isolation of compounds
